# Overcoming immunotherapy resistance in gastric cancer: insights into mechanisms and emerging strategies

**DOI:** 10.1038/s41419-025-07385-7

**Published:** 2025-02-07

**Authors:** Dingtian Luo, Jing Zhou, Shuiliang Ruan, Binzhong Zhang, Huali Zhu, Yangming Que, Shijie Ying, Xiaowen Li, Yuanmin Hu, Zhengwei Song

**Affiliations:** 1 Gastroenterology Department, the Second Affiliated Hospital of Jiaxing University, Jiaxing, Zhejiang China; 2 Department of Surgery, the Second Affiliated Hospital of Jiaxing University, Jiaxing, Zhejiang China; 3 Pathology Department, the Second Affiliated Hospital of Jiaxing University, Jiaxing, Zhejiang China; 4 Intensive Care Unit, the Second Affiliated Hospital of Jiaxing University, Jiaxing, Zhejiang China

**Keywords:** Cancer immunotherapy, Tumour immunology

## Abstract

Gastric cancer (GC) remains a leading cause of cancer-related mortality worldwide, with limited treatment options in advanced stages. Immunotherapy, particularly immune checkpoint inhibitors (ICIs) targeting PD1/PD-L1, has emerged as a promising therapeutic approach. However, a significant proportion of patients exhibit primary or acquired resistance, limiting the overall efficacy of immunotherapy. This review provides a comprehensive analysis of the mechanisms underlying immunotherapy resistance in GC, including the role of the tumor immune microenvironment, dynamic PD-L1 expression, compensatory activation of other immune checkpoints, and tumor genomic instability. Furthermore, the review explores GC-specific factors such as molecular subtypes, unique immune evasion mechanisms, and the impact of Helicobacter pylori infection. We also discuss emerging strategies to overcome resistance, including combination therapies, novel immunotherapeutic approaches, and personalized treatment strategies based on tumor genomics and the immune microenvironment. By highlighting these key areas, this review aims to inform future research directions and clinical practice, ultimately improving outcomes for GC patients undergoing immunotherapy.

## FACTS

- Gastric cancer remains a highly lethal disease, with immunotherapy resistance being a major clinical challenge.
- The tumor-immune microenvironment significantly impacts immune checkpoint inhibitor efficacy in gastric cancer.
- Unique molecular and immune subtypes of gastric cancer influence resistance mechanisms and treatment outcomes.
- Combination therapies are emerging as promising strategies to overcome immunotherapy resistance in gastric cancer.

## Open questions

- What are the precise mechanisms by which the tumor-immune microenvironment drives immunotherapy resistance in gastric cancer?
- What are the key signaling pathways involved in ECM remodeling that hinder T-cell infiltration in gastric cancer?
- Can personalized treatment strategies based on gastric cancer molecular subtypes effectively overcome immunotherapy resistance?
- How can novel combination therapies be optimized to enhance the efficacy of immune checkpoint inhibitors in gastric cancer?

## Introduction

GC remains one of the leading causes of cancer-related mortality worldwide, with advanced stages presenting particularly challenging therapeutic scenarios [1–3]. Despite advances in treatment modalities such as surgery, chemotherapy, and radiotherapy, the prognosis for advanced GC patients continues to be poor [1, 4]. Recently, immunotherapy, particularly ICIs targeting PD1/PD-L1, has emerged as a promising approach, offering new hope for improving patient outcomes [5, 6].

ICIs work by blocking the interaction between PD1 on immune cells and PD-L1 on tumor cells, thereby relieving the immune system’s suppression and allowing it to mount an effective antitumor response [7, 8]. In GC, PD1/PD-L1 inhibitors have demonstrated clinical efficacy, especially in patients with specific molecular subtypes [9, 10]. However, the benefits of immunotherapy are not universal. A significant proportion of patients experience either primary resistance, where the therapy fails to elicit a response, or acquired resistance, where the initial response diminishes over time [11, 12].

Understanding the mechanisms underlying immunotherapy resistance in GC is crucial for improving treatment outcomes. Resistance mechanisms are multifaceted, involving alterations in the tumor-immune microenvironment, dynamic changes in PD-L1 expression, compensatory activation of alternative immune checkpoints, and intrinsic tumor genomic instability [13, 14]. Additionally, GC-specific factors, such as distinct molecular subtypes and unique immune evasion mechanisms, including the role of Helicobacter pylori infection, further complicate the landscape of resistance [15].

This review aims to provide a comprehensive overview of the current understanding of immunotherapy resistance in GC. We will explore the intricate mechanisms contributing to resistance, discuss emerging strategies to overcome these barriers and highlight future directions for research and clinical practice. By elucidating these aspects, we hope to offer insights that will guide the development of more effective therapeutic strategies for GC patients undergoing immunotherapy.

## Current status of immunotherapy in GC

The main ICIs currently used in the treatment of GC include PD1 inhibitors, PD-L1 inhibitors, and CTLA-4 inhibitors, each with specific indications. PD1 inhibitors such as pembrolizumab and nivolumab are commonly used in GC treatment [16–18]. Pembrolizumab has been approved for multiple cancer types [19, 20] and was first approved by the FDA in 2017 for patients with previously treated GC, especially those with PD-L1-positive tumors (CPS ≥ 1) [21, 22]. It has shown significant efficacy in patients with high microsatellite instability (MSI-H) or mismatch repair deficiency (dMMR) [23]. Nivolumab, another PD1 inhibitor, has been approved for GC in multiple countries. The CheckMate-649 study demonstrated that nivolumab combined with chemotherapy significantly extended overall survival (OS) in untreated advanced GC patients, particularly those with PD-L1 CPS ≥ 5, compared to chemotherapy alone. It is indicated for first-line treatment of advanced or metastatic GC in combination with chemotherapy and as monotherapy for recurrent or metastatic cases [9, 24].

The primary PD-L1 inhibitor studied in GC is atezolizumab, with extensive research focusing on its combination with other treatments. While not yet formally approved by the FDA or EMA for GC, it has shown promising outcomes in some clinical trials [25, 26]. CTLA-4 inhibitors, such as ipilimumab, are approved for melanoma and other cancers. In GC, studies on ipilimumab are still ongoing, often in combination with PD1 inhibitors like nivolumab. However, ipilimumab has not been approved for GC monotherapy, though combination treatments show potential [27, 28].

Multiple phase III trials have confirmed the efficacy and safety of ICIs in GC. The CheckMate-649 trial showed that nivolumab plus chemotherapy significantly improved OS and progression-free survival (PFS), with the most common adverse events being nausea, diarrhea, and peripheral neuropathy [29]. In the ATTRACTION-4 trial, nivolumab combined with oxaliplatin-based chemotherapy significantly improved PFS in Asian patients with HER2-negative, unresectable advanced or recurrent GC, though it did not improve OS. Common adverse events included neutropenia, thrombocytopenia, and decreased appetite [30]. The phase III KEYNOTE-062 trial showed that pembrolizumab monotherapy was non-inferior to chemotherapy in terms of OS for untreated advanced GC or gastroesophageal junction (GEJ) cancer patients with PD-L1 CPS ≥ 1, with fewer adverse events. However, neither pembrolizumab nor its combination with chemotherapy showed superiority in OS or PFS over chemotherapy alone [31]. The KEYNOTE-811 trial demonstrated that pembrolizumab combined with first-line trastuzumab and chemotherapy significantly improved PFS in patients with PD-L1 CPS ≥ 1 metastatic HER2-positive GC, with the most common treatment-related adverse events (TRAEs) being diarrhea, nausea, and anemia [32]. Finally, the KEYNOTE-859 trial indicated that pembrolizumab plus chemotherapy significantly improved OS with manageable toxicity, with the most common grade 3–5 adverse events being anemia and neutropenia [33].

We summarize the clinical trial results of ICIs in GC in Table 1 for a clearer overview.

**Table 1:** The clinical trial results of ICIs in GC.

ClinicalTrials.gov	Drugs	Combination	Results	Ref
*NCT02370498*	Pembrolizumab	/	No significant OS improvement but higher 24-month OS rate vs. paclitaxel. Enhanced OS benefit in PD-L1-positive gastric/GEJ cancer. Fewer TRAEs than paclitaxel.	[222]
NCT02589496	Pembrolizumab	/	~50% resistance to pembrolizumab. Nonsynonymous mutations correlate with antitumor activity and prolonged PFS. Diverse TCR repertoire and increased PD1 + CD8 + T cells linked to longer PFS and durable clinical benefit.	[223]
NCT02335411	Pembrolizumab	/	Promising activity and manageable safety in advanced gastric/GEJ cancer after ≥2 prior lines. Durable response observed in both PD-L1+ and PD-L1− tumors.	[224]
NCT02370498	Pembrolizumab	/	No significant OS improvement in PD-L1+ advanced G/GEJ cancer, but longer DOR and better safety profile.	[225]
NCT01848834	Pembrolizumab	/	Manageable toxicity and promising antitumor activity in PD-L1+ recurrent/metastatic GC; warrants further investigation in Phase II/III trials.	[226]
*NCT02494583*	Pembrolizumab	/	Maintains HRQOL; similar to chemotherapy in this population.	[227]
NCT02589496	Pembrolizumab	/	Pembrolizumab (MSI-H, EBV+ tumors): ORR 85.7% and 100%, respectively. In PD-L1+ (CPS ≥ 1%) vs. PD-L1− patients, ORR significantly higher (50.0% vs. 0.0%, P < 0.001). ctDNA levels at 6 weeks predict response and PFS; ctDNA decline correlates with better outcomes.	[228]
NCT02494583	Pembrolizumab	Chemotherapy	Pembrolizumab (PD-L1 CPS ≥ 1): OS non-inferior to chemotherapy, but not superior. For PD-L1 CPS ≥ 10, pembrolizumab extended OS, though not statistically significant. Pembrolizumab + chemotherapy showed no improvement in OS or PFS vs. chemotherapy alone. Fewer severe TRAEs with pembrolizumab.	[31]
NCT03675737	Pembrolizumab	Chemotherapy	Significantly extended OS, especially in PD-L1 CPS ≥ 1 and CPS ≥ 10 patients.	[33]
NCT02013154	Pembrolizumab	DKN-01	Good tolerability, no new safety signals. Enhanced antitumor activity in DKK1-high tumors in treatment-naive GEJ/GC patients.	[229]
NCT03609359	Pembrolizumab	Lenvatinib	Promising antitumor activity and acceptable safety in advanced GC. Confirmatory trials planned based on these results.	[230]
NCT03019588	Pembrolizumab	Paclitaxel	Inconclusive results due to small sample size, but show good tolerability and similar efficacy trend to KEYNOTE-061.	[231]
NCT02370498	Pembrolizumab	Paclitaxel	Similar HRQoL in advanced gastric and GEJ cancer patients.	[232]
NCT03615326	Pembrolizumab	Trastuzumab and chemotherapy	Trastuzumab + Chemotherapy vs. Pembrolizumab + Trastuzumab + Chemotherapy: Pembrolizumab significantly reduces tumor volume, achieves complete response in some patients, and markedly improves objective response rate.	[233]
NCT03382600	Pembrolizumab	SOX or S-1 and SP	Pembrolizumab + SOX or SP: Shows good efficacy and manageable safety.	[234]
NCT02267343	Nivolumab	/	Significantly improves long-term survival in advanced gastric/GEJ cancer patients.	[235]
NCT02951091	Nivolumab	/	Demonstrates durable survival benefit.	[236]
apicCTI-183895 (ClinicalTrials.jp)	Nivolumab	/	Neoadjuvant Nivolumab (monotherapy): Feasible in some resectable GC patients, with acceptable safety and potential for inducing major pathological responses.	[237]
NCT02872116	Nivolumab	Chemotherapy	Shows stable or improved HRQoL compared to chemotherapy alone in advanced/metastatic non-HER2 + GC /GEJC/EAC, with reduced risk of HRQoL deterioration.	[238]
NCT04078295	Nivolumab	E7389-LF	Shows promising antitumor activity in GC patients, with no new safety signals compared to monotherapy.	[239]
NCT03878472	Camrelizumab	Apatinib and S-1 ± oxaliplatin	Complete Pathological Response Rate: 15.8% Major Pathological Response Rate: 26.3% Pathological Response: Significantly associated with MSI, PD-L1 expression, and TMB.	[240]
NCT04195828	Camrelizumab	Apatinib and chemotherapy	Good tolerability and favorable response in locally advanced GC.	[241]
NCT04345783	Camrelizumab	Apatinib and S-1	Promising antitumor activity and manageable toxicity in advanced gastric/GEJ adenocarcinoma, regardless of PD-L1 expression.	[242]
NCT02734004	Durvalumab	Olaparib	Shows promising antitumor activity and safety.	[243]
*NCT03941873*	Tislelizumab	Sitravatinib	Generally well-tolerated, with preliminary antitumor activity in advanced HCC and GC /GEJC patients.	[244]
NCT05844371	Tislelizumab	XELOX therapy	Adding PD1 inhibitor improves 1-year DFS rate in locally lymph node-positive GC vs. chemotherapy alone. Treatment is safe and well-tolerated.	[245]
NCT02915432	Torpalima	XELOX	Shows manageable safety and promising antitumor activity in AGC patients, especially when combined with XELOX. High TMB may predict OS in AGC patients receiving monotherapy.	[246]
NCT03193190 and NCT03281369	Atezolizumab	PEGPH20	No clinical activity in GC patients. Safety profile consistent with known safety of each drug.	[247]
NCT03667170	Envafolimab	/	As the first subcutaneously administered single-domain anti-PD-L1 antibody, demonstrates good efficacy and acceptable safety in treating advanced dMMR/MSI-H solid tumors, with potential as a significant advancement in cancer therapy.	[248]
NCT01943461	Avelumab	/	Shows acceptable safety in advanced solid tumor patients in Japan, with some clinical activity in advanced gastric/GEJ cancer patients.	[249]
NCT04182724	PD1 inhibitor (selected according to the patient’s needs)	Albumin-bound paclitaxel and apatinib	Shows certain efficacy and safety in mGC patients.	[250]
NCT02699515	Bintrafusp (a first-in-class bifunctional fusion protein, composed of the extracellular domain of the TGFβ RII receptor fused to a human IgG1 antibody targeting PD-L1)	/	Shows controllable safety and clinical activity.	[251]
NCT03710265	SHR-1701 (a novel bifunctional fusion protein, composed of an anti-PD-L1 monoclonal antibody fused with the extracellular domain of TGF-β receptor II)	/	Demonstrates good safety and encouraging antitumor activity, providing a basis for further exploration.	[252]

## Mechanisms of immunotherapy resistance in GC

**Fig. 1:**
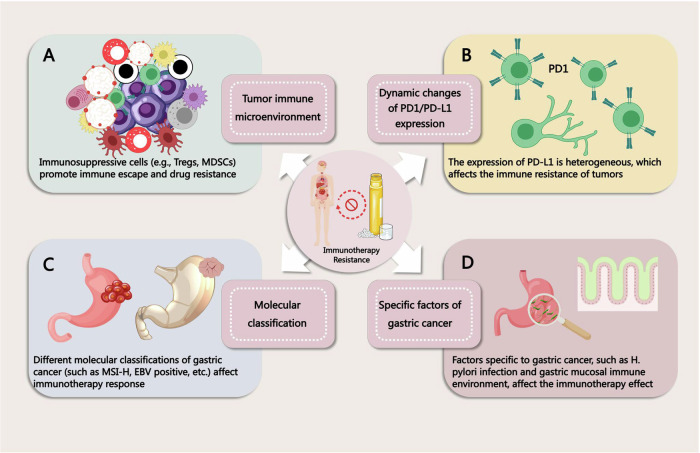
Factors Contributing to Immunotherapy Resistance in GC. **A** Immunosuppressive tumor microenvironments (such as Tregs and MDSCs) promote immune escape and drug resistance in gastric cancer. **B** The heterogeneity of PD1/PD-L1 expression in tumor and TME affects the response of gastric cancer to immunotherapy. **C** Different molecular classifications (such as MSI-H, EBV positive, etc.) and subtypes of gastric cancer affect the efficacy of immunotherapy. **D** Factors specific to gastric cancer, such as H. pylori infection and gastric mucosal immune environment, affect the efficacy of immunotherapy.

Multiple factors contribute to the complex process of immunotherapy resistance in GC. The tumor microenvironment (TME) weakens the antitumor-immune response through the interactions of immunosuppressive cells and signaling pathways, while the dynamic changes in PD-L1 expression exacerbate resistance to ICIs. The molecular classification of GC reveals differences in immunotherapy responses across subtypes, and unique immune evasion mechanisms further drive the development of resistance. (Fig. 1).

### Tumor-immune microenvironment

The tumor-immune microenvironment in GC is a complex and dynamic network of cells, signaling molecules, and extracellular matrix components that interact with tumor cells and play a critical role in modulating the immune response. The composition of the TME includes immune cells such as T lymphocytes, natural killer (NK) cells, dendritic cells (DCs), myeloid-derived suppressor cells (MDSCs), regulatory T cells (Tregs), and tumor-associated macrophages (TAMs), alongside fibroblasts and endothelial cells. While certain immune cells such as cytotoxic T cells and NK cells contribute to antitumor immunity, immunosuppressive components within the TME can promote immune evasion and resistance to therapies, including immunotherapy.

**Fig. 2:**
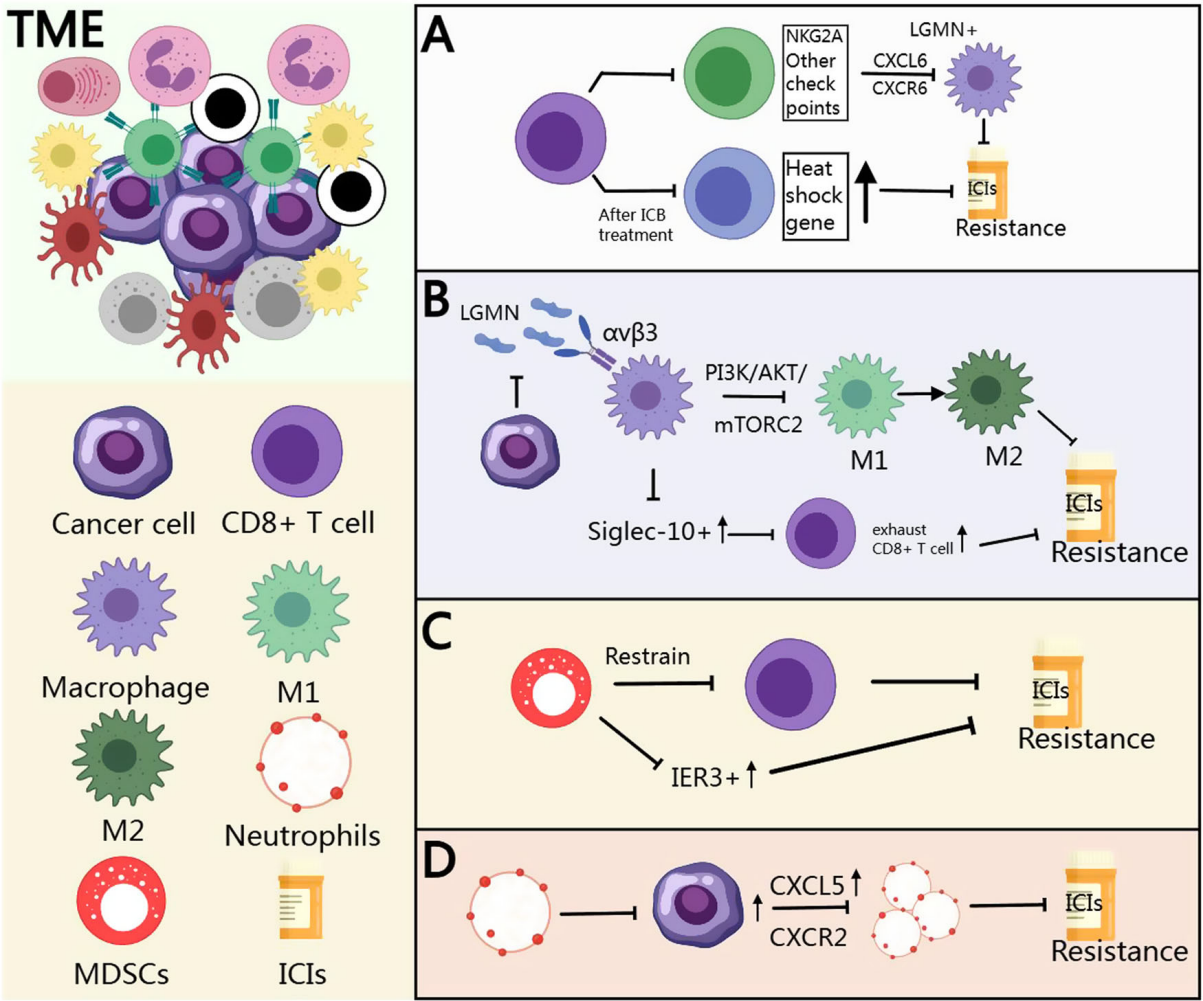
Mechanisms of Immune Cells in the TME Contributing to Immunotherapy Resistance. **A** Mechanisms of T cell involvement in immunotherapy resistance in gastric cancer. **B** Mechanisms of TAM involvement in immunotherapy resistance. **C** Mechanisms of MDSCs involvement in immunotherapy resistance. **D** Mechanisms of neutrophils involvement in immunotherapy resistance.

Cytotoxic T cells are key effectors in the antitumor-immune response, directly targeting and killing cancer cells. However, in the GC TME, their activity is often suppressed by immunosuppressive factors, leading to reduced effectiveness and contributing to immune evasion (Fig. 2A). Research has identified two distinct immune checkpoint expression patterns (ICEP1 and ICEP2) in GC. ICEP1 includes CD8 + T cells co-expressing PD1, CTLA-4, TIGIT, LAG-3, or CD38, while ICEP2 involves CD8 + T cells expressing NKG2A alone or co-expressing it with other checkpoints. The ICEP2 subgroup is associated with resistance to anti-PD1 therapy in GC, potentially mediated by the recruitment of LGMN+ macrophages via the CXCL16-CXCR6 signaling pathway [34]. Additionally, another study found that heat shock gene expression in intratumoral CD4/CD8 + T cells was significantly upregulated following immune checkpoint blockade (ICB) therapy, particularly in non-responsive tumors, suggesting that stress response T cells characterized by heat shock gene expression may be linked to immunotherapy resistance [35].

**Fig. 3:**
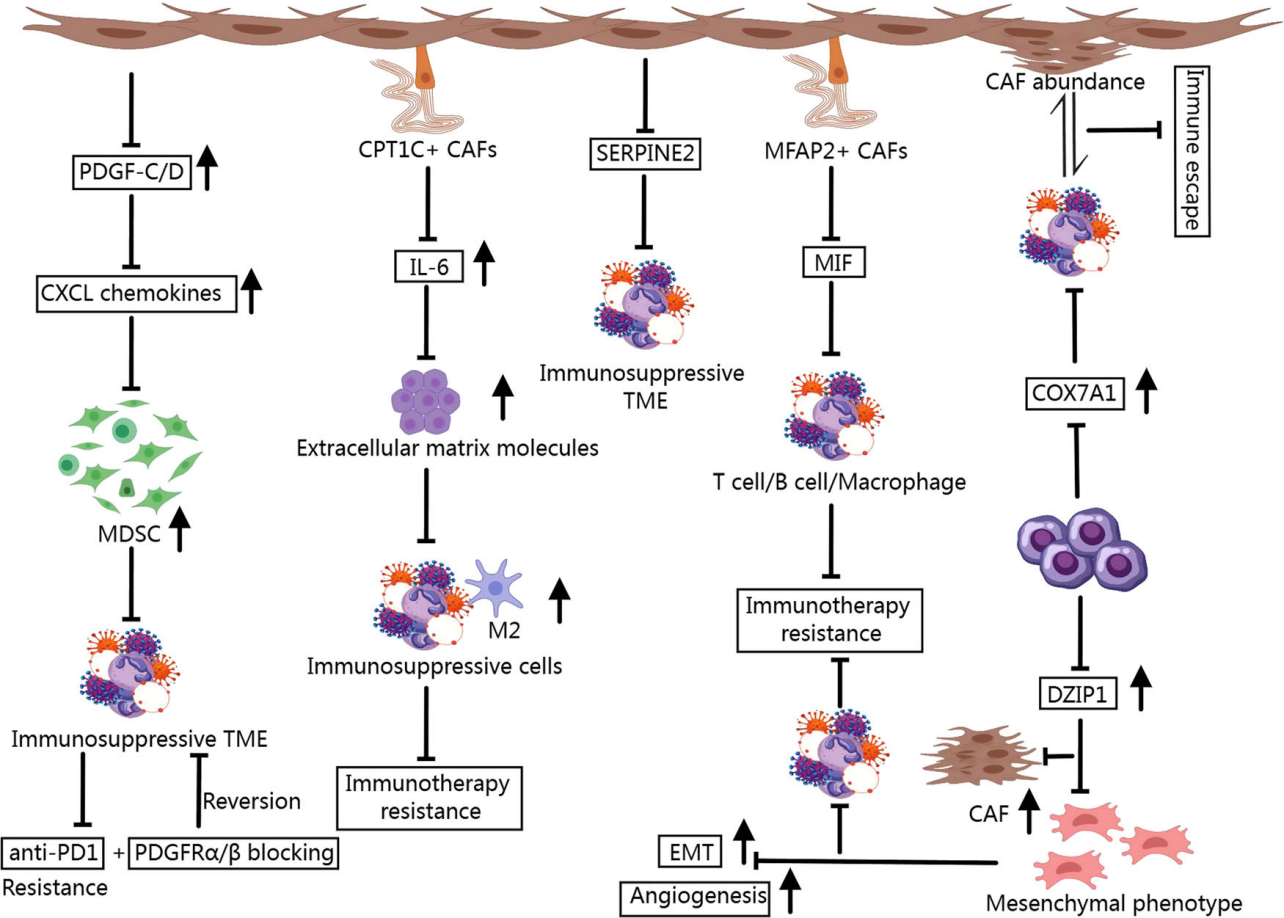
The role of CAFs in promoting immunotherapy resistance in GC and potential therapeutic targets to overcome resistance. CAFs contribute to an immunosuppressive tumor microenvironment through various mechanisms, including secretion of immunosuppressive factors, recruitment of immunosuppressive cells, and modulation of immune checkpoint signaling. Targeting key CAF-associated pathways, such as the PDGF-C/D and IL-6 signaling axes, may enhance the efficacy of immunotherapy and reverse resistance in gastric cancer.

Cancer-associated fibroblasts (CAFs), as key components of the TME, exhibit significant heterogeneity and can be classified into distinct subtypes based on their phenotypic and functional characteristics. These subtypes play diverse roles in tumor progression and immune modulation, significantly influencing the efficacy of immunotherapy [36, 37]. The main subtypes include myofibroblast CAFs (myCAFs), inflammatory CAFs (iCAFs), and antigen-presenting CAFs (apCAFs), among others [38, 39]. MyCAFs are characterized by high α-SMA expression and are primarily involved in ECM remodeling [40, 41]. These cells enhance tissue stiffness, create physical barriers to immune cell infiltration, and contribute to tumor invasion [42, 43]. ICAFs exhibit low α-SMA expression and secrete pro-inflammatory cytokines such as IL-6 and CXCL1 [41, 44]. They promote an immunosuppressive TME by recruiting MDSCs and inhibiting T-cell activation [45, 46]. In GC, ICAFs may contribute to immune evasion and resistance to ICIs. Single-cell study has shown that iCAFs can interact with T cells by secreting IL-6 and CXCL12. iCAFs not only showed enhanced pro-invasion activity but also mobilized surrounding immune cells to build a microenvironment favorable to tumors. Therefore, inhibiting their activation inhibits the GC “seeds” while improving the GC soil [47]. ApCAFs express MHC II molecules and exhibit antigen-presenting potential [48, 49]. They might modulate T-cell responses [50]; however, their role remains controversial and requires further investigation. Although less studied in gastric cancer, their immunomodulatory role could influence response to immunotherapy. In addition, a study has shown that CAFs in GC promote immune evasion via the PDGF-C/D signaling pathway, specifically by recruiting suppressive myeloid cells through the increased expression of CXCL chemokines, leading to anti-PD1 therapy resistance. Blocking PDGFRα/β can reverse the immunosuppressive TME by remodeling the tumor stroma and, when combined with anti-PD1 therapy, synergistically suppresses the growth of fibrotic tumors [51]. A specific CAF subset in GC—CPT1C+ CAFs—promotes immunosuppression in the TME by secreting IL-6, enriching extracellular matrix molecules, and recruiting immunosuppressive cells, particularly M2 macrophages. High levels of CPT1C+ CAFs are associated with poor response to immunotherapy in GC patients [52]. Furthermore, multiple models utilizing fibroblast-associated gene markers have been developed to predict immunotherapy response, providing new avenues for overcoming immune resistance in GC [53–55]. Figure 3 illustrates some of the roles of CAFs in immunotherapy for GC.

TAMs often exhibit an immunosuppressive M2 phenotype in GC [56, 57]. They promote tumor growth and immune evasion, contributing to resistance to immunotherapy (Fig. 2B). Studies have revealed that GC cells secrete legumain (LGMN), which binds to integrin αvβ3 on macrophages, activating the PI3K/AKT/mTORC2 signaling pathway and driving the polarization of macrophages from an M1 to M2 phenotype, thereby promoting immune evasion and resistance to anti-PD1 therapy. Blocking LGMN or integrin αvβ3 can significantly inhibit this process [58]. Additionally, high levels of Siglec-10+ TAMs promote CD8 + T cell exhaustion, contributing to immune evasion and chemotherapy resistance. Blocking Siglec-10 reactivates antitumor immunity and shows synergistic effects when combined with anti-PD1 therapy [59].

MDSCs suppress T-cell activity and create an immunosuppressive environment, contributing to resistance to immunotherapy in GC [60] (Fig. 2C). Studies have shown that in the GC TME, infiltrating monocytic MDSCs (M-MDSCs) highly express immunosuppressive genes and are significantly enriched in GC tissues, with IER3 + M-MDSCs being closely associated with immunosuppression and treatment resistance [61].

Neutrophils in the TME can promote tumor growth and suppress antitumor immunity, contributing to immunotherapy resistance in GC (Fig. 2D). In anti-PD1 therapy for GC, overexpression of CXCL5 recruits tumor-associated neutrophils via the CXCL5/CXCR2 axis, which is a key factor in driving immunosuppression. Apatinib blocks this process, enhancing the effectiveness of anti-PD1 therapy [62].

In addition to the above, DCs and NK cells also play potential roles in immunotherapy resistance in GC [63–65]. In Fig. 1, we present a schematic of the immune microenvironment associated with immunotherapy resistance in GC for a clearer visualization.

The ECM has also been shown to be an important factor affecting the invasion and function of immune cells in tumors and the effect of immunotherapy [66, 67]. The ECM is a complex network of proteins, glycoproteins, and proteoglycans that provide structural and biochemical support to cells [68]. In GC, ECM undergoes significant remodeling, promotes tumor progression and immune escape, and plays a crucial role in regulating the infiltration of immune cells, especially T cells, thus affecting the efficacy of immunotherapy [47, 69]. Studies have shown that TRIM44 is highly expressed in gastric cancer and is associated with T-cell infiltration. TRIM44 inhibits gastric tumorigenicity by regulating T-cell-mediated antitumor immunity and LOXL2 protein levels. Mechanically, TRIM44 directly binds to LOXL2, affecting the stability of LOXL2, altering extracellular matrix remodeling, and affecting tumor immunity [70]. Key ECM components, such as collagen [71], laminin [72], and fibronectin [73], are often overexpressed in GC, leading to increased matrix stiffness and [74, 75]. This enhanced rigidity not only restricts the physical movement of T cells into the tumor core but also alters integrin-mediated signaling pathways, reducing the ability of T cells to adhere to and migrate through the ECM [76]. Additionally, the ECM can sequester immunosuppressive molecules like TGF-β, further suppressing T-cell activation and infiltration [77]. CAFs, which are abundant in GC, play a pivotal role in ECM remodeling. CAFs deposit excessive ECM components and promote crosslinking of collagen fibers via lysyl oxidase, exacerbating matrix stiffness [78]. ECM remodeling in cancer is driven by dynamic interactions between tumor cells, stromal cells, and soluble factors [79]. Matrix metalloproteinases are key enzymes that degrade ECM components, facilitating tumor invasion and altering immune cell accessibility [80]. However, paradoxically, excessive ECM degradation can release bioactive fragments called matrikines, which may promote tumor growth and immunosuppression [81]. In addition, the accumulation of hyaluronic acid and fibronectin in the extracellular matrix are also important factors affecting the tumor-immune microenvironment [82, 83]. In conclusion, in a variety of tumors, including gastric cancer, ECM is a key barrier to effective immunotherapy, primarily by limiting T-cell invasion and inducing an immunosuppressive environment. Understanding the mechanisms of ECM remodeling and its effects on immune cell dynamics is critical to developing new therapeutic strategies. By combining ECM-targeted therapy with existing immunotherapies, it is possible to enhance the immune response and improve outcomes in GC patients.

### Dynamic changes of PD-L1 expression

The dynamic and heterogeneous expression of PD-L1 in GC plays a critical role in mediating resistance to immunotherapy. PD-L1 expression can vary spatially and temporally within the TME, influenced by intrinsic tumor factors and external signals such as inflammation and immune cell interactions. This heterogeneity complicates the prediction of therapeutic response to PD1/PD-L1 inhibitors, as fluctuating levels of PD-L1 can lead to inconsistent responses to treatment. Understanding the mechanisms driving these dynamic changes in PD-L1 expression is crucial for overcoming resistance and improving the efficacy of immunotherapy in GC.

A study analyzing 1014 GC specimens through immunohistochemistry evaluated the clinical significance of PD1 and its ligands (PD-L1 and PD-L2) in GC. The results showed that PD-L1 was expressed in 37.8% of tumor cell membranes and 74.9% of infiltrating immune cells. Higher PD-L1 expression was observed in patients without metastasis, Epstein-Barr virus (EBV)-positive patients, and those with elevated C-met and PCNA expression. Additionally, patients with high PD-L1 expression exhibited better survival rates. Increased PD-L1, PD-L2, and PD1 expression was found in patients with higher T-cell infiltration, which may be linked to adaptive immune resistance mechanisms [84].

Previous studies have shown that GC mesenchymal stem cells (MSCs) promote PD-L1 expression and lactate production via the IL-8/CXCR2 pathway, impairing the antitumor efficacy of PD1 immunotherapy. Blocking the IL-8/CXCR2 pathway or reducing PD-L1 expression and lactate production significantly restored the antitumor effects of PD1 antibodies [85]. Similar findings revealed that GC MSCs enhanced PD-L1 expression in GC cells, leading to resistance to CD8 + T-cell cytotoxicity. This resistance was mediated by IL-8 released from GC MSCs, which activated STAT3 and mTOR signaling pathways, promoting c-Myc induction and increasing PD-L1 expression in GC cells. Consistently, blocking IL-8 helped overcome immune evasion and improved the efficiency of immunotherapy [86].

Certain microRNAs, such as miR-105-5p, can inhibit post-transcriptional PD-L1 expression by binding to a key cis-acting element in the PD-L1 3’ untranslated region. This reduces PD-L1 protein and surface expression, promoting CD8 + T-cell activation. The expression of miR-105-5p is regulated by DNA methylation of its host gene GABRA3 promoter [87]. Additionally, all-trans retinoic acid (ATRA) enhances PD-L1 expression by increasing its protein stability and synthesis. ATRA-induced PD-L1 upregulation strongly confers resistance to activated T-cell cytotoxicity in GC cells and antagonizes the effects of PD-L1 antibodies [88].

### Molecular classification and immunotherapy resistance

In recent years, the molecular classification of GC has provided valuable insights into the mechanisms of immunotherapy resistance, helping to identify distinct tumor subtypes with varying responses to ICIs. These classifications are based on genetic, epigenetic, and immune profiling, offering a more precise understanding of tumor heterogeneity and its role in immune evasion. Different molecular subtypes exhibit varying levels of sensitivity to immunotherapy [89–91]. For example, based on the characteristics of immune cell infiltration and their functional states, it can be classified into inflamed, immune-excluded, and immune-desert subtypes [92–94]. The inflamed subtype is characterized by high levels of T-cell and NK cell infiltration, active interferon signaling, and frequent PD-L1 overexpression, which generally makes it sensitive to ICIs [95]. The immune-excluded subtype, although containing immune cells, is marked by their confinement to the tumor periphery, potentially due to barriers imposed by CAFs and the ECM, leading to resistance to ICIs [94, 96–98]. The immune-desert subtype shows minimal immune cell infiltration, typically associated with low tumor immunogenicity and immune evasion mechanisms, and represents a prototypical feature of resistance to ICIs [99, 100]. These subtypes also significantly affect the response of gastric cancer to immunotherapy. Understanding these molecular classifications is crucial for developing more effective strategies to overcome resistance and tailor immunotherapy approaches to individual patients with GC.

A study explored the efficacy of neoadjuvant immunotherapy with nivolumab and ipilimumab in patients with resectable dMMR/MSI-H gastric or GEJ adenocarcinoma. The findings demonstrated the potential efficacy and safety of this immunotherapy regimen in this subset of gastric/GEJ adenocarcinoma [101]. Similarly, studies have shown that tumors with MSI-H tend to be resistant to chemotherapy but may exhibit durable responses to immunotherapy. In some cases, EBV-positive patients achieved complete long-term responses to immunotherapy [102]. However, dMMR/MSI-H gastrointestinal cancers with peritoneal metastasis and ascites respond poorly to ICIs [103]. A high number of mutations in the PI3K-AKT-mTOR pathway (NMP) genes may predict primary resistance to ICIs in dMMR/MSI-H gastric adenocarcinoma, and the use of PI3K-AKT-mTOR inhibitors as adjuncts to immunotherapy is recommended for patients with high NMP mutations [104]. Nonetheless, real-world cases suggest that MMR status and microsatellite stability may not fully predict GC resistance to anti-PD1 therapy [105].

For HER2-positive patients, studies indicate a good objective response rate to ICI treatment. The efficacy of ICIs in patients with liver metastases from GC is associated with peritoneal metastasis status, and HER2-positive patients may derive greater clinical benefit [106]. In metastatic/unresectable HER2-negative GC patients, those with a higher relative abundance of Lactobacillus exhibited better responses to immunotherapy and longer PFS, suggesting that Lactobacillus may serve as a novel adjuvant to enhance the efficacy of immunotherapy in GC [107].

Beyond the traditional classifications, some studies have reclassified GC patients to provide guidance for immunotherapy and precision medicine [108–116], such as the immune-inflamed, immune-excluded, and immune-desert phenotypes mentioned above. In immune-desert GC, epithelial-mesenchymal transition (EMT) signaling is highly enriched, rendering these tumors insensitive to CTLA-4 blockade [117]. Another study utilized a sample-specific edge perturbation matrix based on global immune gene network backgrounds to identify four molecular network subtypes of GC (MNG). Among these, MNG-1 exhibited the best prognosis with robust cell cycle activity, while MNG-2 was enriched for the immune-hot phenotype, showing potential responsiveness to immunotherapy. MNG-3 and MNG-4 were associated with EMT and had poorer prognoses. Notably, MNG-4 displayed chromosomal instability and an immune-desert microenvironment, showing a propensity for metastasis and resistance to immunotherapy [118].

Additionally, several studies have developed predictive models to classify GC patients based on their immunotherapy outcomes, helping to predict prognosis and treatment response [119–121]. For instance, the epigenetic modification disorder score, characterized by high FTO expression and low HDAC1 expression, showed features of immune suppression [122]; the immunogenic cell death-related gene risk score (ICDRS), where patients with low ICDRS had better prognoses and were more sensitive to immunotherapy [123]; the DNA damage repair (DDR) signature score, where patients with low DDR signature scores may not benefit from adjuvant chemotherapy or anti-PD1 monoclonal antibody treatment [124]; the ICI score system, where a low ICI score was associated with increased tumor mutational burden (TMB) and served as a potential prognostic and predictive biomarker for chemotherapy and immunotherapy [125]; and the stromal score, where patients with low stromal scores had higher TMB and MSI, making them more sensitive to PD1/PD-L1 ICIs. Conversely, high stromal score subtypes exhibited activation of transforming growth factors and EMT, potentially leading to T-cell suppression and resistance to immunotherapy [126].

### The role of gut microbiota in immunotherapy resistance in GC

The gut microbiota has emerged as a crucial factor in modulating immune responses and significantly influencing the efficacy of immunotherapy [127, 128]. In the context of GC, dysbiosis (microbial imbalance) can impact the tumor-immune microenvironment, contributing to resistance to ICIs [129, 130]. Specific microbial signatures have been shown to influence the effectiveness of ICIs by modulating T-cell function, antigen presentation, and immune checkpoint expression [131]. Gut microbes such as *Bacteroides fragilis* and *Faecalibacterium prausnitzii* have been associated with a robust T-cell response. These microbes promote the differentiation of CD4+ and CD8 + T cells into effector T cells, enhancing antitumor immunity [132, 133]. On the other hand, dysbiosis, characterized by an overgrowth of bacteria like *Enterococcus faecalis* or *Fusobacterium nucleatum*, may impair T-cell activation and reduce T-cell infiltration into the tumor, contributing to immunotherapy resistance [134, 135].

In GC, helicobacter pylori (HP) infection upregulates the expression of CD80 and CD86 in gastric epithelial cells and activates T-cell response [136]. In addition, previous studies have shown that HP inhibits the proliferation of CD4 + T cells and reduces the synthesis of IL-2 and IFN-g by upregulating the expression of PD-L1 on gastric epithelial cells [137, 138]. It has also been shown that HP infection can induce IgA production by B cells by activating Group 2 innate lymphocytes [139]. In addition, Methylbacterium in gastric cancer tissue inhibited CD8+ tissue-resident memory T cells in TME while limiting TGF-b expression [140]. A retrospective study showed that Stenotrophomonas and Selenomonas were positively associated with BDCA2+ plasmacytoid DC (pDC) and Foxp3+ Treg. Comamonas is negatively correlated with BDCA2+ pDC, which is involved in the immune escape of GC cells [141]. In addition to the gut microbiota itself, its metabolites also play an important role in cancer immunity. Studies have shown that intestinal microbial metabolites can regulate immune cell phenotype and function by regulating the secretion of immunosuppressive cytokines [142]. These metabolites can enhance immune cell function by binding to immune cells [143–145]. For example, studies have shown that short chain fatty acids (SCFAs) can maintain intestinal homeostasis by promoting IL-10 production in Th1 cells [144, 146, 147]. Another study has shown that SCFAs inhibits histone deacetylase by binding to GPR41, and promotes the production of IL22 by CD4 + T cells, thereby inhibiting inflammation [148].

In addition to regulating tumor immunity, intestinal flora can affect the efficacy of tumor immunotherapy. Choi et al. found that ICB therapy induces an enhanced antitumor-immune response by metastasizing to secondary lymphatic organs of tumors and intestinal bacteria such as bifidobacterium, streptococcus, and Lactobacillus [149]. Several studies have confirmed that the microbiome and its metabolites may have a broad impact on anti-gastric cancer immunotherapy mediated by cytokine secretion and enhanced T-cell infiltration [150, 151]. GC can be divided into four types: EBV positive, MSI, genomic stability, and chromosomal instability [152]. GC large-scale microbiota profiles from two demographically distinct cohorts showed that Selenoides, Bacteroides, and porphyromonas were the top three microorganisms in MSI high GC patients [153]. In addition, in addition to high levels of MSI and EBV-positive status, HP infection is not only an indicator of high PD-L1 expression but also an indicator of poor prognosis after immunotherapy [138, 154]. This may be a predictor of immunotherapy efficacy in GC patients.

## Strategies to overcome immunotherapy resistance

### Targeting potential therapeutic resistance pathways

Overcoming immunotherapy resistance in GC requires a precise approach that targets specific molecular pathways contributing to immune evasion. Several potential targets have been identified, including immune checkpoint molecules, immunosuppressive cells, and signaling pathways involved in the TME. By focusing on these key pathways, it is possible to modulate the immune response and improve the efficacy of existing treatments. Identifying and inhibiting these resistance mechanisms holds promise for improving patient outcomes and overcoming the limitations of current immunotherapies.

Several classical tumor targets also play crucial roles in immunotherapy resistance in GC. VISTA, predominantly expressed on TAMs, is linked to poor clinical prognosis and reduced response to immunotherapy. In GC, VISTA+ TAMs exhibit a mixed phenotype that impairs CD8 + T-cell function. Blocking VISTA can reprogram TAMs into a pro-inflammatory state, thereby reactivating CD8 + T cells, promoting tumor cell apoptosis, and enhancing the efficacy of PD1 inhibitors [155]. The loss of Smad4 in GC confers an immune evasion advantage. Unlike their Smad4-expressing counterparts, Smad4-deficient gastric organoids form tumors in immunocompetent mice. These GC cells secrete CXCL1, inhibiting DC differentiation and promoting granulocytic MDSC (G-MDSC) accumulation, while also enhancing CD133+ cancer stem cell-like populations. Moreover, Smad4 deficiency upregulates PD-L1 expression and downregulates 4-1BBL, leading to immune evasion. Dual checkpoint blockade with anti-PD-L1 and anti-CTLA-4 antibodies or treatment with agonistic anti-4-1BB antibodies effectively targets Smad4-deficient xenografts [156]. Elevated VCAN expression is associated with poor prognosis in GC and resistance to immunotherapy. Patients with low VCAN expression benefit more from adjuvant chemotherapy and radiotherapy. High VCAN expression correlates with increased CAF infiltration and enrichment of stroma-related pathways, suggesting VCAN is a promising biomarker for predicting treatment response [157].

In GC, CAFs secrete SERPINE2, promoting an immunosuppressive microenvironment and contributing to immune evasion and treatment resistance. Targeting CAF-derived SERPINE2 could be a potential strategy to overcome immunotherapy resistance [158]. Additionally, MFAP2+ CAFs, through the release of macrophage migration inhibitory factors, influence T cells, B cells, and macrophages to create an immunosuppressive environment, further promoting treatment resistance. These findings highlight the potential of MFAP2+ CAFs as therapeutic targets in GC [159]. Overexpression of COX7A1 in GC regulates fibroblast abundance and communication with immune cells, inducing immune evasion. Monitoring COX7A1 expression may help predict prognosis, chemotherapy resistance, and immunotherapy outcomes [160]. Furthermore, DAZ-interacting zinc finger protein 1 (DZIP1) is upregulated in both CAFs and malignant epithelial cells in GC and is strongly associated with the mesenchymal phenotype. DZIP1 promotes CAF proliferation and enhances EMT in GC cells, driving angiogenesis and invasion. It is also linked to immunosuppressive TME, leading to poor responses to immunotherapy, making DZIP1 a potential target for overcoming resistance [161] (Fig. 3).

Notably, ATRX mutations are more frequent in female GC patients than in males. Female patients with ATRX mutations exhibit higher MSI, TMB, and PD-L1 expression, as well as increased anti-cancer immune indicators such as IFN-γ signaling, cytolytic activity, and antigen-presentation machinery scores. ATRX mutations may enhance immunogenicity by affecting DDR pathways, suggesting that ATRX could serve as a potential predictive biomarker for ICI therapy in female GC patients [162].

A comprehensive summary of additional potential targets to enhance immunotherapy sensitivity in GC is provided in Table 2 for easier reference.

**Table 2:** Potential targets to enhance immunotherapy sensitivity in GC.

Targets	Mechanism	Clinical Translation	Ref
IL-8/CXCR2	GC MSCs promote PD-L1 expression and lactate production through the IL-8/CXCR2 pathway, weakening the antitumor effects of anti-PD1 immunotherapy.	Blocking the IL-8/CXCR2 pathway can significantly restore the antitumor efficacy of anti-PD1 antibodies.	[85]
miR-105-5p	miR-105-5p binds to PD-L1 and inhibits its post-transcriptional expression, reducing both PD-L1 protein and surface levels, thereby promoting CD8 + T-cell activation.	It may serve as a potential biomarker and target for PD1/PD-L1 therapy.	[87]
ATRA	ATRA enhances PD-L1 expression by increasing its protein stability and synthesis.	RUX inhibits ATRA-induced PD-L1 expression and resensitizes GC cells to anti-PD-L1 therapy.	[88]
IL-8	IL-8 released by GC MSCs activates the STAT3 and mTOR signaling pathways, promoting c-Myc induction and thereby increasing PD-L1 expression in GC cells.	Blocking IL-8 may help overcome immune evasion in GC cells and enhance the efficacy of immunotherapy.	[86]
SERPINE2	CAFs promote the formation of an immunosuppressive microenvironment by secreting the protein SERPINE2, leading to increased immune evasion and resistance to immunotherapy in tumors.	SERPINE2 is a promising immuno-oncology target with potential for combination with immunotherapy to enhance treatment efficacy.	[158]
GAS6/AXL	The GAS6/AXL pathway plays a role in inhibiting T-cell activation. Knocking out GAS6 or using the AXL inhibitor CCB-3233 can reduce the expression of immunosuppressive genes and increase the infiltration of tumor-infiltrating T cells.	Targeting the GAS6/AXL pathway may be a new strategy to overcome immunotherapy resistance in GC.	[253]
MFAP2	MFAP2+ CAFs create an immunosuppressive environment by releasing MIF, which affects T cells, B cells, and macrophages, thereby promoting therapeutic resistance.	Immunosuppressive MFAP2+ CAFs have potential as therapeutic targets in GC.	[159]
PLEK2	PLEK2 promotes Sp1 phosphorylation through the PI3K-AKT pathway, leading to the upregulation of MT1-MMP expression and the shedding of MICA.	PLEK2 inhibits NK cell immune surveillance by promoting the shedding of MICA, making MICA shedding a potential therapeutic target for GC.	[254]
Smad4	Smad4-deficient GC cells suppress DC differentiation and promote G-MDSC accumulation through CXCL1 secretion. Additionally, Smad4 deficiency increases PD-L1 expression and decreases 4-1BBL expression, leading to immunogenic changes and the expansion of CD133 + CSC-like cells.	The findings support the use of ICB therapy in patients with advanced GC exhibiting Smad4 loss.	[156]
VCAN	High VCAN expression is associated with increased resistance to immunotherapy, while low VCAN expression correlates with better outcomes from adjuvant chemotherapy and chemoradiotherapy. High VCAN tumors show increased fibroblast infiltration and enriched matrix-related signaling pathways.	VCAN is a promising biomarker for predicting responses to various treatments in GC patients.	[157]
DZIP1	DZIP1 directly promotes CAF proliferation and enhances EMT in GC cells, driving angiogenesis. It is associated with the immune-suppressive microenvironment in GC, leading to poor responses to immunotherapy.	DZIP1 may serve as a potential therapeutic target for GC.	[161]
VISTA	VISTA is primarily expressed on TAMs and is associated with poorer clinical outcomes and reduced response to immunotherapy. VISTA+ TAMs exhibit a mixed phenotype that impairs CD8 + T-cell function. Blocking VISTA can shift TAMs to a pro-inflammatory phenotype, thereby reactivating CD8 + T cells, promoting tumor cell apoptosis, and enhancing the efficacy of PD1 inhibitors.	VISTA plays a crucial role in immunotherapy resistance in GC, and blocking VISTA may synergistically enhance the efficacy of PD1 inhibitors.	[155]
IL-4	IL-4 induces metabolic changes in macrophages, activating the PI3K/AKT/mTOR pathway, which increases ATP production and glycolysis, leading to elevated lactate generation and upregulation of FcγRIIB expression. These changes ultimately result in CD8 + T-cell dysfunction and resistance to PD1 antibody therapy.	IL-4 may be a potential target for improving GC treatment outcomes.	[255]
IL-1R1	IL-1R1 contributes to the formation of an immunosuppressive microenvironment by promoting M2 macrophage polarization and reducing CD8 + T-cell infiltration.	IL-1R1 can serve as a biomarker for predicting treatment outcomes in GC, and IL-1R1 antagonists may represent a new therapeutic strategy.	[256]
ATXN2	ATXN2 promotes chemotherapy resistance in GC by activating the PI3K/AKT pathway and increasing BCL2L1 expression. Additionally, ATXN2 stimulates PD-L1 expression, enhancing immune treatment efficacy. SP1 has been found to regulate ATXN2 expression through transcriptional control, thereby influencing chemotherapy resistance and immune evasion in GC.	The study highlights the critical roles of the SP1/ATXN2/PI3K-AKT/BCL2L1 and SP1/ATXN2/PI3K-AKT/PD-L1 signaling pathways in GC, providing new theoretical and experimental insights for overcoming chemotherapy resistance and enhancing the efficacy of immune therapies.	[257]
RIPK2	RIPK2 promotes immune therapy resistance through the IL-6/JAK/STAT3 signaling pathway and the interferon-γ and interferon-α response pathways.	RIPK2 may serve as a prognostic biomarker and contribute to immune therapy resistance by inducing dysfunction in cytotoxic T lymphocytes.	[258]
APB	GC tumors with high APB levels exhibit immune exhaustion characteristics and poor responses to ICIs, with significantly shorter PFS compared to patients with low APB levels.	Targeting APB may be a potential strategy to improve treatment outcomes in GC.	[259]
NOTCH3	High expression of NOTCH3 is associated with reduced infiltration of CD8 + T cells and increased presence of immunosuppressive cells in the TME. Elevated NOTCH3 levels are linked to increased expression of ICIs and weakened immune responses. Furthermore, NOTCH3 expression is negatively correlated with predictive biomarkers for ICB immunotherapy.	These findings reveal the role of NOTCH3 in immune evasion in GC and suggest its potential as a therapeutic target or predictive biomarker.	[260]
ANTXR1	ANTXR1 may mediate immune suppression by promoting the secretion of immune-suppressive factors, which play crucial roles in regulating tumor-associated fibroblast transformation, M2 macrophage polarization, and T-cell exhaustion.	ANTXR1 may serve as a prognostic biomarker and a potential target for immune therapy in GC.	[261]
HIGD1B	HIGD1B is associated with cancer progression and late-stage pathways. High expression of HIGD1B in patients is linked to increased tumor-infiltrating immune cells after chemotherapy and immunotherapy, contributing to immune evasion and resistance.	HIGD1B is a promising biomarker and potential therapeutic target.	[262]
MFG-E8	MFG-E8 is highly expressed in GC tissues and cells, and its expression positively correlates with M2 macrophage infiltration. MFG-E8 may play a role in the cAMP signaling pathway. Patients with high MFG-E8 expression are more prone to treatment resistance.	MFG-E8 may serve as a prognostic marker and a potential target for immune therapy in GC.	[263]
LGMN	LGMN promotes immune evasion and resistance to anti-PD1 therapy in GC by binding to integrin αvβ3 on the cell surface and activating the PI3K/AKT/mTORC2 signaling pathway, leading to the polarization of macrophages from an M1 to an M2 phenotype.	Blocking LGMN or integrin αv expression can significantly inhibit this process, suggesting that the sLGMN/integrin αvβ3/PI3K/AKT/mTORC2 axis could be a novel therapeutic target for GC.	[58]
CXCL5	Overexpression of CXCL5 recruits TANs to the TME through the CXCL5/CXCR2 axis, which is a key factor contributing to immune suppression.	Targeting the CXCL5/CXCR2 axis has shown promising results in models, indicating its potential as a therapeutic target to overcome immune therapy resistance.	[62]
Siglec-10	Siglec-10+ TAMs exert immunosuppressive effects in GC by promoting CD8 + T-cell exhaustion, leading to immune evasion. Blocking Siglec-10 can reactivate antitumor-immune responses and shows synergistic effects when combined with anti-PD1 therapy.	Targeting Siglec-10 represents a promising strategy for immune therapy in GC.	[59]
PDGF-C/D	CAFs promote immune evasion through the PDGF-C/D signaling pathway, particularly by increasing the expression of CXCL family chemokines, which recruit PMN-MDSCs and contribute to resistance to anti-PD1 therapy.	Blocking PDGFRα/β can reverse the immunosuppressive microenvironment by modulating the tumor stroma. When used in combination with anti-PD1 therapy, it synergistically inhibits the growth of fibrotic tumors, highlighting the potential of stromal reprogramming in enhancing cancer immunotherapy.	[51]
IER3	IER3 + M-MDSCs are closely associated with immune suppression and treatment resistance. The presence of IER3 + M-MDSCs is strongly linked to poor prognosis in GC patients, particularly evident in those with treatment resistance.	These cells may play a central role in the development of immune suppression and ICI resistance within the GC TME.	[61]
B7H7	B7H7-silenced DCs were more effective than untransfected DCs in inducing T-cell proliferation and altering the cytokine profile of T cells, such as increased IL-4 and reduced TGF-β.	Inhibiting B7H7 in DCs can enhance their stimulatory effect on T cells, potentially improving the efficacy of cancer immunotherapy.	[65]
CPT1C	CPT1C+ CAFs promote the conversion of macrophages to the M2 phenotype and are a major source of IL-6, which is closely associated with M2 phenotype formation. Inhibiting CPT1C expression significantly reduces IL-6 secretion.	CPT1C+ CAFs induce M2 macrophages by secreting IL-6, which impairs antitumor immunity in GC, providing new insights into the immune suppression mechanisms in GC.	[52]

### Combination therapies

While ICIs have shown significant therapeutic potential, many patients experience limited or short-lived responses due to the complexity of tumor-immune interactions and the presence of immunosuppressive mechanisms within the TME. To address these challenges, combination approaches that incorporate ICIs with other therapeutic modalities, such as chemotherapy, targeted therapies, radiation, or additional immune-modulating agents, have been explored. These combinations aim to enhance antitumor immunity, overcome intrinsic and acquired resistance, and ultimately improve clinical outcomes. By targeting multiple pathways simultaneously, combination therapies have the potential to convert immunologically “cold” tumors into “hot” ones, thereby increasing the likelihood of a sustained response to immunotherapy [163].

Chemotherapy has been shown to have an immunomodulatory effect, enhancing tumor immunogenicity by promoting immunogenic cell death and increasing the release of tumor antigens [164–166]. Studies have demonstrated that combining PD1/PD-L1 inhibitors with chemotherapeutic agents like fluoropyrimidine, oxaliplatin, and irinotecan improves immune recognition by upregulating MHC-I expression and reducing tumor-associated immunosuppressive cells, such as MDSCs and Tregs. For instance, in the KEYNOTE-062 trial, pembrolizumab combined with chemotherapy showed promising results in improving the overall response rate and PFS in advanced GC patients [167–171]. The clinical trials of ICIs combined with chemotherapy in GC are summarized in Table 1.

In the previous section, we discussed the molecular targets associated with ICI resistance in GC. Targeted therapies against these pathways can modulate the TME and enhance immune responses. For instance, anti-HER2 drugs combined with immunotherapy significantly increase the infiltration of NK cells, CD8 + T cells, and B lymphocytes in GC. In responsive patients, the interactions between these cells are strengthened, particularly through the CCL3/CCL4-CCR5 signaling pathway, where NK cells recruit CD8 + T cells. Meanwhile, B lymphocytes interact with M2 macrophages and Tregs via multiple signaling pathways, inhibiting immune resistance [64]. Similarly, anti-VEGF drugs like ramucirumab normalize aberrant tumor vasculature, improving T-cell infiltration and reducing the immunosuppressive environment. The combination of ramucirumab with pembrolizumab has been explored in several studies [3]. Relevant clinical trials of ICIs combined with targeted therapies are summarized in Table 1.

Anti-angiogenic therapies that target VEGF pathways have been shown to enhance the efficacy of ICIs by reducing the immunosuppressive effects of TME. VEGF not only promotes tumor angiogenesis but also impairs immune cell trafficking and promotes Treg and MDSC infiltration. By combining VEGF inhibitors with ICIs, the normalization of blood vessels can enhance the immune system’s ability to access the tumor [172–174]. Trials such as the REGONIVO study, which combined regorafenib (a multi-kinase inhibitor) with nivolumab (a PD1 inhibitor), demonstrated promising activity in heavily pretreated GC patients, suggesting that anti-angiogenic agents may restore immune surveillance and improve ICI efficacy [175].

Radiotherapy can induce a systemic immune response known as the “abscopal effect,” where localized radiation leads to the destruction of distant, non-irradiated tumor sites through immune-mediated mechanisms [176, 177]. In GC, radiotherapy has been shown to increase the release of tumor-associated antigens and enhance antigen presentation, thus sensitizing tumors to ICIs. The combination of radiation and ICIs is currently being explored in clinical settings, with early results showing enhanced antitumor effects through increased T-cell activation and inhibition of immune-suppressive pathways [178–180].

Epigenetic alterations are key drivers of immune resistance in cancer. Agents targeting epigenetic modifications, such as DNA methyltransferase inhibitors and histone deacetylase inhibitors, can reprogram the TME to become more immunogenic [181–183]. For example, HDAC inhibitors can enhance the expression of immune-related genes, promote the activity of NK cells, and increase tumor antigen presentation [181]. Combining HDAC inhibitors like vorinostat or romidepsin with PD1 inhibitors has shown preclinical promise, offering a rationale for clinical trials to test their efficacy in reversing resistance to ICIs [184, 185].

Dual ICI therapy is another promising combination approach [186]. The combination of anti-PD1 and anti-CTLA-4 therapies can increase the infiltration of tumor-specific CD8 + T cells, although many of these T cells exhibit an exhausted phenotype. Studies have shown that in resistant tumors, abnormal activation of the JAK-STAT pathway is observed, along with infiltration of macrophages, neutrophils, and Tregs. Introducing JAK inhibitors has been shown to restore CD8 + T-cell function and reshape the immunosuppressive TME, further enhancing the efficacy of dual ICI therapy [187]. Additionally, a clinical case reported complete remission in a HER2-positive advanced GEJ cancer patient through dual PD1/CTLA-4 bispecific immunotherapy combined with chemotherapy, providing a novel and effective treatment option for HER2-positive patients. This approach should be considered as an alternative when trastuzumab is not feasible [188].

### Emerging immunotherapy

Emerging therapeutic strategies aim to develop novel immunotherapy approaches or integrate innovative techniques within existing frameworks to enhance the response of GC patients to immunotherapy. In recent years, several promising cutting-edge strategies have rapidly evolved, particularly in the regulation of antitumor-immune responses and personalized treatment.

Cell-based therapies, such as chimeric antigen receptor T-cell (CAR-T) therapy, have achieved significant success in hematologic malignancies and are now being extended to solid tumors. For GC, CAR-T cells targeting specific antigens like CLDN18.2 and MET are under development and testing [189–193]. Additionally, new immune effector cell therapies, such as CAR-NK cell therapy, have demonstrated favorable safety profiles and preclinical efficacy [194, 195]. Compared to CAR-T cells, CAR-NK cells offer advantages such as lower toxicity and the lack of need for matched donor sources. Specifically, in the TME of GC, CAR-NK cells enhance immune cell infiltration and antitumor activity.

The gut microbiota plays a crucial role in host immune responses and is closely linked to the efficacy of ICIs [149, 196, 197]. Studies have shown that the abundance of certain gut microbes positively correlates with the response of GC patients to ICIs. For instance, the increased presence of Lactobacillus and Bifidobacterium is associated with improved efficacy of PD1/PD-L1 inhibitors [107, 198–200]. By modulating the gut microbiota through methods such as probiotics, fecal transplantation, or selective amplification of specific microbial populations, the TME can be improved, thereby enhancing the sensitivity of patients to immunotherapy [129, 201, 202]. These microbiota-based therapies have entered clinical trials and may become an important component of GC immunotherapy in the future [203, 204].

RNA vaccine technology, such as mRNA vaccines, has garnered attention due to the successful development of COVID-19 vaccines [205, 206]. Beyond infectious disease prevention, mRNA vaccines hold great potential in cancer immunotherapy [207, 208]. Neoantigen vaccines for GC can be personalized based on specific mutations or antigens in the patient’s tumor, designed to trigger a stronger antitumor-immune response [209, 210]. This personalized vaccine strategy enhances T-cell recognition of GC-specific antigens and promotes the generation of memory T cells, thereby improving long-term immune surveillance. Moreover, mRNA vaccines can be combined with ICIs to maximize their therapeutic benefits [211, 212].

The regulation of metabolic pathways within the TME also affects the efficacy of immunotherapy. Studies have found that metabolic competition in the immunosuppressive TME, such as lactate accumulation and glucose depletion, inhibits the function of effector T cells [213–215]. By using metabolic modulators, such as inhibitors of lactate dehydrogenase or glucose transporter protein, the metabolic state of the TME can be reprogrammed, restoring T-cell antitumor activity [216, 217]. Furthermore, regulating mitochondrial function in tumor cells is also considered a potential approach to enhancing the efficacy of immunotherapy [218, 219].

Additionally, advances in CRISPR-Cas9 technology offer new possibilities for GC treatment, especially in enhancing immunotherapy sensitivity and overcoming resistance. Through gene editing, inhibitory receptors like PD1 can be knocked out in T cells, or resistance-related genes can be knocked out in tumor cells, thereby improving responsiveness to immunotherapy [220, 221]. These emerging therapeutic strategies provide new opportunities to overcome resistance to GC immunotherapy. As technology continues to advance, frontier areas such as cell therapy, gene editing, gut microbiota modulation, RNA vaccines, and metabolic regulation will further expand the applications of GC immunotherapy and provide more evidence for individualized treatment approaches.

## Future perspectives and challenges

Despite notable progress with ICIs in the treatment of GC, several limitations persist. First, the heterogeneity of GC leads to significant variability in patient responses to immunotherapy, and current biomarkers are insufficient for effectively predicting therapeutic outcomes. Most studies focus on PD-L1 expression levels, but this singular marker does not adequately explain the complex mechanisms of resistance. Moreover, immune-suppressive factors within the TME, such as TAMs, Treg, and metabolite accumulation, have not been thoroughly investigated, hindering our understanding of their role in ICI resistance. These gaps in knowledge obstruct the development of more precise immunotherapeutic strategies.

Future research should prioritize uncovering new resistance mechanisms and biomarkers. For instance, besides PD-L1, other immune evasion pathways—such as tumor neoantigen burden, metabolic pathway abnormalities, and gut microbiome influences—should be the focus of investigation. Integrating multi-omics data, including genomics, transcriptomics, metabolomics, and single-cell sequencing technologies, could help identify additional key molecules associated with immunotherapy sensitivity and resistance.

Optimizing combination therapies is also a crucial future direction. While dual ICI therapies targeting PD1/PD-L1 and CTLA-4 have shown potential, issues related to toxicity and tolerability remain unresolved. Future studies should explore combinations with metabolic inhibitors, gut microbiome modulators, or agents targeting the TME to enhance the efficacy of immunotherapy.

Translating laboratory research findings into clinical applications presents several challenges. Clinical trial designs and patient recruitment must account for GC’s heterogeneity and treatment response variations among different subtypes. Although preclinical models have demonstrated the potential of combination therapies, their effectiveness, and safety in actual patients require validation through large-scale clinical trials. Developing universal protocols that can be applied to the majority of patients remains challenging due to the individualized nature of immunotherapy.

Furthermore, toxicity management is a critical challenge in the clinical translation of immunotherapy. Particularly with dual ICB or combined with other therapies, the risk of adverse effects complicates clinical application. Future clinical research needs to focus on balancing efficacy with safety, and exploring safer and more effective treatment combinations and dosing regimens.

In summary, future research must address resistance mechanisms in GC immunotherapy, refine personalized combination treatment strategies, and overcome translational obstacles from laboratory to clinical practice. Despite the challenges, ongoing innovation and multidisciplinary collaboration hold promise for advancing the effectiveness of immunotherapy for GC patients.

## Conclusion

In conclusion, overcoming immunotherapy resistance in GC requires a multifaceted approach. While ICIs have made strides in treatment, significant challenges remain, including tumor heterogeneity and insufficient biomarkers for predicting response. Future research should focus on identifying novel resistance mechanisms, exploring new biomarkers, and optimizing combination therapies to enhance efficacy. Translating these findings into clinical practice presents additional hurdles, such as managing toxicity and designing effective clinical trials. Nonetheless, continued innovation and collaborative efforts are essential to advancing therapeutic strategies and improving outcomes for GC patients.
